# Outcomes and microbiological patterns of bacteremia in chemotherapy-related febrile neutropenia at a tertiary facility in Kenya

**DOI:** 10.1016/j.ijregi.2025.100708

**Published:** 2025-07-18

**Authors:** Abhijit Ghose, Jasmit Shah, Anne Mwirigi, Charles Makasa, Felix Riunga

**Affiliations:** 1 Department of Medicine, Aga Khan University, Nairobi, Kenya; 2 Brain and Mind Institute, Aga Khan University, Nairobi, Kenya; 3 Department of Haemato-Oncology, Aga Khan University, Nairobi, Kenya

**Keywords:** Febrile neutropenia, Kenya, Outcomes, Microbiological patterns, Sub-Saharan Africa

## Abstract

**Introduction:**

Febrile neutropenia (FN) is a major cause of mortality and morbidity in patients with malignancy post-chmotherapy. Current guidelines for managing FN recommend starting empiric antibiotic therapy (EAT) promptly. The choice of antibiotic is based on the patient’s condition, local data on common microorganisms isolated, and their resistance patterns. Of note, there is a growing trend of isolating multi-drug–resistant gram-negative bacteria in these patients. In sub-Saharan Africa, there are insufficient data on the mortality rates of FN. There is also a lack of local epidemiologic data on the frequently isolated microorganisms and their antibiotic resistance patterns. This makes it challenging to determine the appropriate EAT for patients with FN. This study aimed to determine the mortality rates of FN in patients with cancer and characterize the isolated microorganisms and their resistance patterns.

**Methods:**

This was a retrospective descriptive study of adult patients with cancer hospitalized with FN post-chemotherapy between January 2017 and July 2022 at Aga Khan University Hospital, Kenya. Continuous variables are expressed as medians with inter-quartile ranges whereas categorical variables are expressed as frequencies and percentages.

**Results:**

A total of 90 episodes of FN involving 50 unique patients were identified from medical records. The median age was 42.8 years (inter-quartile range: 31.3-65.0). Male patients accounted for 67.0% (n = 60). Hematologic malignancies accounted for 90.0% (n = 81). The mortality rate was 26.0% (n = 13), with 84.6% (n = 11) having a hematologic malignancy. Of the 90 episodes, 27.8% (25) had positive blood cultures; 68.0% (17) were gram-negative, 24.0% (six) were gram-positive, and 8.0% (two) were candida species. *Klebsiella pneumoniae* was the most common bacteria isolated. Among gram-negative bacteremia, 23.5% (four) were third-generation cephalosporin-resistant and 11.7% (two) were carbapenem-resistant.

**Conclusions:**

The study found a higher mortality rate in patients with FN in East Africa than in North America. Gram-negative bacteria were the most commonly isolated pathogens in blood cultures, with a growing concern over the increasing prevalence of multi-drug–resistant strains. Further multi-centered studies involving larger sample sizes are required to help develop regional guidelines for the choice of EAT in the management of FN.

## Highlights

• Higher mortality rates were found in patients with febrile neutropenia in East Africa.
• Gram-negative bacteria are more commonly isolated in blood.
• There are concerns for increasing isolation of multi-drug–resistant bacteria.

## Introduction

Febrile neutropenia (FN) is a significant and potentially life-threatening complication among patients with cancer receiving chemotherapy [1]. Chemotherapy remains one of the cornerstones in the treatment of cancer and plays a key role in the effective treatment of cancer because studies have demonstrated an increase in tumor cell burden reduction with an increase in chemotherapy dosage [2]. FN episodes may result in delays or reductions in chemotherapy dosing, which can compromise the efficacy of cancer treatment.

Current management guidelines for FN include a detailed history, physical examination, and appropriate laboratory and imaging investigations to determine the source and severity of the infection. Patients are then risk-stratified using prognostication scores, such as the Multinational Association for Supportive Care in Cancer score, to determine whether they require inpatient or outpatient care. The patients are then initiated on empirical antibiotic therapy [1,3]. The selection of empiric antibiotic therapy (EAT) is determined by the local antibiotic resistance patterns of pathogens and patient factors such as the source of infection, severity of infection, and presence of indwelling devices such as central venous lines [3].

Over the past decades, the microbial etiology of FN has evolved. Although gram-negative bacteria predominated during the early years of chemotherapy use in the 1960s, gram-positive organisms became more common in the early 2000s, largely due to the widespread use of intravenous catheters [[4], [5], [6], [7]]. More recently, gram-negative organisms have re-emerged worldwide, often demonstrating resistance to multiple antibiotics. This has led to a growing reliance on broad-spectrum agents, such as β-lactam antibiotics with antipseudomonal activity, as preferred empiric therapy [3,8,9]. However, increasing resistance to these antibiotics has raised concerns about the overuse of broad-spectrum agents and the resulting selection of multi-drug–resistant organisms [3,5,8]. This results in the need for broader EAT. Overuse of broad-spectrum antibiotics fuels the growth of resistant organisms, perpetuating a vicious cycle. There is, therefore, a need for appropriate choice of antibiotics, as well as timely de-escalation, where appropriate [3].

In high-income countries, large-scale studies have documented FN-related mortality rates around 9.5%, with average hospital stays exceeding 11 days [10]. A unique challenge faced by sub-Saharan Africa (SSA) in the management of chemotherapy-related FN is the lack of data on mortality rates and length of stay. There are a few studies that have looked at children with cancer, such as the multi-center prospective supportive care for children with cancer in Africa (SUCCOUR) study conducted in five sub-Saharan African countries (Zimbabwe, Cameroon, Kenya, Ghana, and Malawi) from September 2019 to March 2020. It looked at 104 FN episodes among children aged <16 years with cancer and reported a mortality of 11.0% [11]. A retrospective study in Ethiopia reported a mortality rate of 20.0% in pediatric patients with cancer with FN, although their sample size was only 60 [12].

Another significant challenge faced in the SSA region is the scarcity of local data on the microbiological etiology and antibiotic resistance patterns of organisms isolated in FN to guide the choice of EAT. The choice of EAT is primarily based on European and North American data. Of the few, one conducted in Uganda looking at causes of bacteremia in febrile patients with cancer demonstrated 24 cases of bacteremia from 170 cases of febrile cancer, with gram-negative bacteria (66.7%) being the most common isolates [13]. It is worth noting that the study used different cutoffs for FN and the study population included adolescents and adults. Local antibiotic resistance patterns are fundamental for appropriate choices in EAT to reduce length of stay (LOS), mortality rates, and incidence of multi-drug–resistant organisms [5,14]. To the best of our knowledge, no studies have been conducted in East Africa to describe the mortality rate or LOS of adult patients with chemotherapy-induced FN. The aim of this study was to determine the mortality rates of FN in patients with cancer and characterize the isolated microorganisms and their resistance patterns.

## Methods

### Study design and participants

This was a retrospective descriptive study involving adult patients admitted with chemotherapy-induced FN between January 1, 2017 and July 31, 2022. The study was conducted at the Aga Khan University Hospital (AKUH), Nairobi, Kenya. The hospital is a 300-bed, private, not-for-profit, tertiary level teaching and referral hospital with a dedicated oncology unit. In addition, the hospital has over 50 outreach centers throughout East Africa providing primary and specialized care and various diagnostic tests.

We included patients aged 18 years and above who had a confirmed absolute neutrophil count of ≤0.5 × 10⁹/l or an anticipated decline to that level and who had received chemotherapy within the preceding 30 days. Eligible patients also had a documented underlying malignancy. We excluded patients with FN due to causes other than recent chemotherapy and those with missing or incomplete data.

Ethical approval was obtained from Institutional Scientific and Research Ethics Committee (ISERC) at the Aga Khan University before conducting the study (Approval Number 2022/ISERC-77). A waiver of consent was sought and provided from the ISERC because this study involved a review of retrospective data. A research license was obtained from the National Commission for Science, Technology, and Innovation (License No: NACOSTI/P/23/23752).

### Data collection and analysis

Data were obtained from the medical records department for all the patients with FN who met the study criteria. The FN episodes were identified using either a discharge or death diagnosis of neutropenia (International Classification of Diseases, Tenth Revision diagnosis code D70.1) combined with a documentation of fever in the patient medical records. One FN episode was considered as a single admission for any patient that met the criteria for febrile neutropenia.

Data obtained included demographic profiles (sex, date of birth), dates of admission and discharge/death, comorbid conditions including specific malignancy, outcome of patient (discharge/death), laboratory values (neutrophil count at admission, blood cultures, organisms isolated, and resistance patterns), number of chemotherapy cycles preceding the FN episode, time elapsed between the last chemotherapy cycle and the FN episode and treatment details (choice of EAT, change in antibiotics, duration of treatment, initiation of antifungals). The resistant patterns for bacteria isolated in blood were classified as the following: pan-susceptible: bacteria that are susceptible to all routinely used first-line antibiotics used for that organism; third-generation cephalosporin-resistant: gram-negative bacteria resistant to ceftriaxone and/or cefotaxime; and carbapenem-resistant: gram-negative bacteria resistant to meropenem or imipenem.

Data were collected using the REDCap software based on an abstraction tool. Records with missing data regarding the variables of interest were excluded. The data were reviewed by an infectious disease specialist to verify that all isolated microorganisms were pathogenic. The REDCap data were stored in the institutional server under the guidance of institutional policies. The data were de-identified for analysis purposes. SPSS was used for data analysis (IBM Statistical Package for the Social Sciences version 22.00).

Categorical data were presented as percentages and frequencies whereas continuous data were presented as medians and interquartile ranges (IQRs). Univariate analysis was used for sub-analyses on group comparisons, whereas the Fisher’s exact test or chi-square was used for categorical data and the Mann–Whitney test or Student’s *t*-test was used for continuous data. A *P* <0.05 was considered statistically significant.

## Results

A total of 132 patient charts with at least one episode of FN were screened. Six patients without an underlying malignancy were excluded. Of the remaining 126 episodes, 27 records were incomplete and nine were missing, leaving 90 episodes of chemotherapy-related FN involving 50 unique patients. The highest number of FN episodes for a single patient was eight.

### Demographic and clinical characteristics

The median age of patients experiencing FN episodes was 42.8 years (IQR 31.3-65.0) and males represented 66.7% of those with FN episodes. Hematologic malignancies were the most common type of cancer observed, accounting for 90.0% (n = 81) of the episodes, with acute myeloid leukemia being the most prevalent malignancy at 43.3% (n = 39). The median LOS for FN episodes was 9 days (IQR 5.0-17.0). The median absolute neutrophil count at admission was 0.17 × 10^9^ /l (IQR 0.02-0.41). Table 1 summarizes the demographic and clinical characteristics of the FN episodes and patients.

**Table 1:** Demographic and clinical characteristics of FN episodes and patients.

Age (years) (median [IQR])		42.84 (31.33, 65.01)	
**Gender**	Male	60	66.7%
**Malignancy**	Solid tumor	9	10.0%
	Hematological	81	90.0%
**Solid tumor**	Breast	2	2.2%
	Cholangiocarcinoma	1	1.1%
	Gastric	1	1.1%
	Glioblastoma multiforme	1	1.1%
	Pancreas	1	1.1%
	Rectal	3	3.3%
**Hematological**	Acute lymphoblastic leukemia	4	4.4%
	Acute myeloid leukemia	39	43.3%
	Chronic lymphocytic leukemia	5	5.6%
	Non-Hodgkin’s lymphoma	27	30.0%
	Multiple myeloma	2	2.2%
	Myelodysplastic syndrome	2	2.2%
**Time elapsed between last chemotherapy cycle and onset of FN episode**	More than 7 days	63	70.0%
**ANC at admission** (× 10^9^/l) (median [IQR])		0.17 [0.02, 0.41]	
**Ongoing chemotherapy protocol** (n = 90)	Highly myeloablative regimen	35	38.9%
	Moderately myeloablative regimen	41	45.6%
	Low myeloablative regimen	14	15.6%
**Number of chemotherapy cycles preceding the FN episode** (n = 90)	<3	45	50.0%
	≥3	41	45.6%
	Continuous chemotherapy	4	4.4%
**Number of previous chemotherapy cycles** (n = 86) (median [IQR])		2.0 [1.0, 5.0]	
**Comorbid conditions** (n = 50)	Yes	25	50.0%
**Comorbid conditions** (n = 25)	Diabetes	5	10.0%
	Hypertension	9	18.0%
	Chronic kidney disease	1	2.0%
	HIV	6	12.0%
	Heart failure or coronary artery disease	4	8.0%
	Others	13	26.0%
**Other comorbid conditions** (n = 13)	Anxiety disorder	1	2.0%
	COVID-19	1	2.0%
	Venous thromboembolism	2	4.0%
	Hepatitis B	2	4.0%
	Hypothyroidism	1	2.0%
	Invasive aspergillosis	4	8.0%
	Psoriatic arthritis	1	2.0%
	Seizure disorder	1	2.0%
**Length of stay** (Days) (median [IQR])		9.0 (5.0, 17.0)	

ANC, absolute neutrophil count; FN, febrile neutropenia; IQR, interquartile range.

The overall in-hospital mortality rate was 26.0% (n = 13 of 50). Among the mortalities, 84.6% (n = 11 of 13) had a hematologic malignancy, with non-Hodgkin’s lymphoma being the most common malignancy at 46.2% (n = 6 of 13). The study found that the deceased patients were older, mostly male (76.9%), and more likely to have a bloodstream infection (BSI) (46.2%) than the discharged patients. However, these values were not statistically significant (*P* >0.05). Table 2 compares the demographic and clinical characteristics between discharged and mortality groups.

**Table 2:** Demographic and clinical characteristics compared between discharged and mortality groups.

		Outcome (n = 90)				*P*-value
		Discharged		Mortality		
		(n = 77)		(n = 13)		
**Age** (years)		40.4 (32.8, 63.6)		55.2 (30.9, 68.3)		0.675
**ANC at admission** (× 10^9^/l) (median [IQR])		0.16 (0.02, 0.37)		0.17 (0.02, 0.42)		0.561
**Gender**	Male	50	64.9%	10	76.9%	0.532
	Female	27	35.1%	3	23.1%	
**Malignancy**	Solid tumor	7	9.1%	2	15.4%	0.657
	Hematological	70	91.0%	11	84.6%	
**Time elapsed between last chemotherapy cycle and onset of febrile neutropenia episode**	Less/Equal to 7 days	25	32.5%	2	15.4%	0.329
	More than 7 days	52	67.5%	11	84.6%	
**Positive blood culture**	Yes	19	24.7%	6	46.2%	0.177
	No	58	75.3%	7	53.8%	

ANC, absolute neutrophil count.

### Microbiological etiology and resistance patterns of bloodstream infection

Blood cultures were collected at the beginning of all the FN episodes. Organisms were isolated in blood in 27.8% (n = 25 of 90) of cases. For these BSI, 68% (n = 17 of 25) were gram-negative rods, 24% (n = 6 of 25) were gram-positive cocci, and 8% (n = 2 of 25) were candidemia. *Klebsiella pneumoniae* was the most commonly isolated bacteria among the gram-negative bacteria at 35.3% (n = 6 of 17). There were six gram-positive BSIs with two *Streptococci* species, two *Enterococci*, one coagulase-negative *Staphylococcus* (CONS), and one case of *Staphylococcus aureus*.

Among the gram-negative organisms isolated, four (23.5%) were third-generation cephalosporin-resistant and two (11.7%) were carbapenem-resistant. A total of 10 (58.8%) were pan-susceptible. One organism, *Haemophilus influenzae*, did not have its resistance pattern reported. The CONS isolated was vancomycin-susceptible. The *S. aureus* isolated was methicillin-susceptible. One of the *Enterococci* species was vancomycin-susceptible and the other was vancomycin-resistant but susceptible to linezolid. The *Streptococci* were penicillin-susceptible. There were two cases of fungemia with *Candida auris.* One of these cases had four repeat admissions with FN; however, the patient was noted to have infective endocarditis, and, therefore, only the initial admission was considered in our study. Table 3 below demonstrates the organisms isolated in blood and their associated antibiotic resistance patterns.

**Table 3:** Microbiological etiology and resistance patterns in bloodstream infection.

**Blood cultures taken at onset of febrile neutropeni**a (n = 90)	Yes	90	100.0%
**Positive blood culture**	Yes	25	27.8%
	No	65	72.2%
**Organism isolated in blood** (n = 25)	Gram positive Cocci	6	24.0%
	Gram negative rods	17	68.0%
	Fungi	2	8.0%
**Gram positive Cocci in blood** (n = 6)	Coagulase negative staphylococcus	1	16.7%
	*Staphylococcus aureus*	1	16.7%
	*Streptococci spp.*	2	33.3%
	*Enterococci spp.*	2	33.3%
**Gram negative rods in blood** (n = 17)	*Pseudomonas aeruginosa*	3	17.6%
	*Escherichia coli*	5	29.4%
	*Klebsiella pneumoniae*	6	35.3%
	Others^a^	3	17.6%
**Resistance pattern of *S. aureus***	Methicillin susceptible *Staphylococcal aureus*	1	100.0%
**Resistance pattern of Streptococci**	Penicillin susceptible	2	100.0%
**Resistance pattern of Enterococci**	Vancomycin-susceptible	1	100.0%
	Vancomycin-resistant	1	100.0%
**Resistance pattern of *P. aeruginosa***	Pan susceptible	3	100.0%
**Resistance pattern of *E. coli***	Third gen cephalosporin-resistant	2	40.0%
	Carbapenem-resistant	2	40.0%
	Pan susceptible	1	20.0%
**Resistance pattern of *K. pneumoniae***	Third gen cephalosporin-resistant	2	33.3%
	Pan susceptible	4	66.7%
**Resistance pattern of other gram-negative bacteria**	Pan susceptible	2	66.7%
	Not reported	1	33. 3%

a Others include *Acinetobacter hemolyticus, Klebsiella oxytoca, Haemophilus influenzae.*

### Treatment patterns for FN episodes

The most common EAT was a carbapenem-based regimen at 85.6% (n = 77 of 90). Monotherapy was preferred in 84.4% (n = 76 of 90) of FN episodes with carbapenems in 65 episodes, piperacillin-tazobactam in six episodes, ceftriaxone in three episodes, and amoxicillin-clavulanate and fluoroquinolone in one each. Combination therapy was used in 15.6% (n = 14 of 90). FN episodes with carbapenem and vancomycin in 11 cases, carbapenem and aminoglycoside in one episode, and the combination of ceftriaxone and azithromycin for two episodes.

A total of 50 (55.6%) of the FN episodes had conversion of antibiotics. The median duration of total antibiotic therapy was 9 days (IQR 7.0-12.0). Among all the FN episodes, 46.7% (n = 42 of 90) received antifungals, of which 76.2% (n = 32 of 42) were empiric therapy. The median day of initiation of empiric antifungals was 3 days (IQR 0.0-5.5) into admission. The most common antifungals used were azoles. Table 4 demonstrates the treatment patterns for the FN episodes.

**Table 4:** Treatment patterns among febrile neutropenic episodes.

**Empiric antibiotic of choice**	Carbapenem	77	85.6%
	Fluoroquinolone	1	1.1%
	Piperacillin/Tazobactam	6	6.7%
	Amoxicillin-clavulanate	1	1.1%
	Aminoglycoside	1	1.1%
	Vancomycin	11	12.2%
	Cephalosporin	5	5.6%
	Azithromycin	2	2.2%
**Conversion to another antibiotic regimen?**	Yes	40	44.4%
	No	50	55.6%
**Duration of total antibiotic therapy** (days) (median [IQR])		9.0 (7.0, 12.0)	
**Did the patient receive antifungals?**	Yes	42	46.7%
	No	48	53.3%
**What was the indication for antifungal therapy?** (n = 42)	Empiric	32	76.2%
	Specific treatment	10	23.8%
**Days into admission for initiation of empiric antifungal** (n = 32) (days) (median [IQR])		3.0 (0.0, 5.5)	

IQR, interquartile range.

## Discussion

Our research indicated that patients admitted to the hospital with chemotherapy-related FN had an overall mortality rate of 26.0%. This is higher than reported in studies conducted in the United States, which reported mortality rates ranging from 7.0% to 10.6% [10,[15], [16], [17], [18], [19]]. When looking at studies conducted in the Middle East, a study in Lebanon had a similar lower mortality rate of 9.3% [5]. In SSA, a study in Accra reported a lower mortality rate of 10.0%, but it had a smaller population of 20 patients with only solid tumors, which could explain the lower mortality rate [20]. One possible explanation for the higher mortality rates in our study could be that AKUH is a tertiary referral facility, which generally receives critically ill patients from the Eastern Africa region. Another possible explanation is the high prevalence of hematologic malignancies among our FN episodes. Studies in Singapore among FN episodes in hematologic disease reported lower mortality rates of 4.7-8.3% [21,22]. However, these studies also included patients with FN with other underlying hematologic conditions such as aplastic anemia and post-stem cell transplant patients, which could explain their lower mortality rates.

Previous studies have found that risk factors for mortality in FN include lung cancer compared with other solid tumors, sepsis, pneumonia, and bilirubin levels [17,23]. In our research, the deceased patients were older, mostly male (76.9%), and more likely to have a BSI (46.2%) than the discharged patients. However, these values were not statistically significant (*P* >0.05). A possible explanation for this could be our small number of mortalities, which may have limited the statistical power.

The median length of stay in our study was 9.0 days (IQR 5.0-17.0), which was longer than previous studies and reviews conducted in the United States, which reported a median LOS of 5 days [10,18]. It is worth noting that these studies had a majority of patients with solid tumors compared with our research which mostly had FN episodes with hematologic malignancies. This difference in patient population could be a possible explanation for the longer LOS demonstrated in our study because hematologic malignancies are associated with prolonged neutropenia. In contrast, a Brazilian prospective cohort study with a majority of patients with hematologic malignancies reported a median LOS of 16 days (IQR 18) [24]. There were minimal data available in SSA in adult patients for comparison.

Blood cultures were obtained at the beginning of each FN episode, per the current guidelines for FN [1,3]. Bacteremia was found in 25.6% [23] of these episodes, consistent with studies conducted in North American and European countries, which ranged from 23.3% to 28.0% [25,26]. However, it was higher than studies conducted in SSA, which ranged from 13.0% to 14.1% [11,13]. The SUCCOUR multi-center study reported a 13.0% rate of bacteremia isolation, possibly due to limited resources because only 15.0% of the cases had blood cultures collected before antibiotic administration. It is worth noting that the SUCCOUR study was conducted among children [11].

A recent global trend seen in patients with cancer with FN is that the prevalence of gram-negative bacteremia has increased. In Spain, Gudiol et al. [4] conducted a study comparing two groups of patients with hematologic malignancies who had BSI during neutropenic episodes. The first group was observed from 1991 to 1996, and the second group was observed from 2000 to 2006. It was found that the etiology of BSIs had shifted in favor of gram-negative organisms for the second group. This may be due to an increase in resistance to fluoroquinolones, resulting from their use for prophylaxis in neutropenia [27,28]. Our research showed that gram-negative bacteria were the most frequently isolated microorganisms in blood, accounting for 68.0% of cases [17]. This is in keeping with recent studies conducted in European and Asian countries such as Italy and South Korea, where gram-negative bacteria were isolated in 57.3-61.9% of BSI cases [27,29]. Our results are also in keeping with studies from SSA and the Middle East in Uganda and Lebanon, respectively, where gram-negative bacteria were isolated from 57.3-66.7% of BSI [5,13].

Another emerging trend is the increasing isolation of multi-drug–resistant gram-negative bacteria in patients with FN worldwide [4,5,13,30]. In this study, 23.5% [4] of the gram-negative bacteria that were isolated in blood were resistant to third-generation cephalosporins. In addition, two 11.7% [2] of the *Escherichia coli* found in blood were found to be carbapenem-resistant. A total of 10 (58.8%) were pan-susceptible to the first-line antibiotics.

According to several studies, CONS and *Streptococci* are the most commonly isolated gram-positive organisms worldwide [4,5,7,13,30]. In our study, we found that only 24.0% [6] of BSIs were caused by gram-positive organisms, with 33.3% [2] of those being *Enterococci* and *Streptococci* species each, and only one case each of CONS and *S. aureus*. The *Streptococci* isolated were penicillin-susceptible. The *S. aureus* was methicillin-sensitive. The CONS isolated was vancomycin-susceptible. Of the two *Enterococci* isolated, one was vancomycin-susceptible and the other was vancomycin-resistant but linezolid-sensitive.

The most common EAT of choice was carbapenems at 85.6% (77) of FN episodes. This is in keeping with the latest guidelines that recommend a β-lactam with antipseudomonal activity as monotherapy or in combination with another antibiotic based on patient risk factors and local resistance patterns [1,3]. Empiric antifungals were on median initiated 3 days (IQR 0.0-5,5) into admission, which was a day earlier than recommended by international guidelines which recommend initiating empiric antifungal therapy if fevers persist in patients with FN after 4-5 days of broad-spectrum antibiotics [1,3].

To the best of our knowledge, this study is the first of its kind in East Africa to provide information on the mortality rate, length of hospital stay, microbiological etiology, and antibiotic resistance patterns among adult patients who experience chemotherapy-related FN. We would like to acknowledge a few limitations. First, AKUH was the only study site, which may not provide a true representation of the larger population because it is a private tertiary referral facility with adequate access to resources. Second, the study’s statistical power may have been limited due to the small sample sizes of bacteremia and mortalities. Lastly, we did not collect data on the use of granulocyte colony-stimulating factor, the presence or absence of indwelling central vascular catheters, and the fever to antibiotic administration time due to challenges with data collection. These are important factors that can influence the outcome of patients.

## Conclusion

The study found a higher mortality rate among patients with chemotherapy-related FN than reported in North America. Gram-negative bacteria were commonly isolated in blood, and there is increasing isolation of multi-drug–resistant gram-negative organisms locally as there is globally. Further studies are required with larger sample sizes and involving multi-center study sites to characterize factors associated with mortality and antibiotic resistance patterns of bacteria isolated in patients with chemotherapy-related FN. This can help develop local guidelines on the choice of EAT.

## Funding

This research did not receive any specific grant from funding agencies in the public, commercial, or not-for-profit sectors.

## Author contributions

A.G. contributed to conceptualisation, writing and revision of the manuscript. J.K. performed data analysis and contributed to manuscript writing. C.M. was responsible for data collection. A.M. assisted with mauscript revision. F.R. contributed to the study’s conceptualisation and participated in writing and critical revision of the manuscript.

## Declarations of competing interest

The authors have no competing interests to declare.

## References

[1] Klastersky J., de Naurois J., Rolston K., Rapoport B., Maschmeyer G., Aapro M. (2016). Management of febrile neutropaenia: ESMO Clinical Practice Guidelines. Ann Oncol.

[2] Frei E., Canellos G.P. (1980). Dose: a critical factor in cancer chemotherapy. Am J Med.

[3] Gudiol C., Aguilar-Guisado M., Azanza J.R., Candel F.J., Cantón R., Carratalà J. (2020). Executive summary of the consensus document of the Spanish Society of Infectious Diseases and Clinical Microbiology (SEIMC), the Spanish Network for Research in Infectious Diseases (REIPI) and the Spanish Society of Haematology and haemotherapy (SEHH) on the management of febrile neutropenia in patients with hematological malignancies. Enferm Infecc Microbiol Clin (Engl).

[4] Gudiol C., Bodro M., Simonetti A., Tubau F., González-Barca E., Cisnal M. (2013). Changing aetiology, clinical features, antimicrobial resistance, and outcomes of bloodstream infection in neutropenic cancer patients. Clin Microbiol Infect.

[5] Moghnieh R., Estaitieh N., Mugharbil A., Jisr T., Abdallah D.I., Ziade F. (2015). Third generation cephalosporin resistant Enterobacteriaceae and multidrug resistant gram-negative bacteria causing bacteremia in febrile neutropenia adult cancer patients in Lebanon, broad spectrum antibiotics use as a major risk factor, and correlation with poor prognosis. Front Cell Infect Microbiol.

[6] Montassier E., Batard E., Gastinne T., Potel G., de La Cochetière M.F. (2013). Recent changes in bacteremia in patients with cancer: a systematic review of epidemiology and antibiotic resistance. Eur J Clin Microbiol Infect Dis.

[7] Wisplinghoff H., Seifert H., Wenzel R.P., Edmond MB. (2003). Current trends in the epidemiology of nosocomial bloodstream infections in patients with hematological malignancies and solid neoplasms in hospitals in the United States. Clin Infect Dis.

[8] Averbuch D., Orasch C., Cordonnier C., Livermore D.M., Mikulska M., Viscoli C. (2013). European guidelines for empirical antibacterial therapy for febrile neutropenic patients in the era of growing resistance: summary of the 2011 4th European Conference on Infections in Leukemia. Haematologica.

[9] Freifeld A.G., Bow E.J., Sepkowitz K.A., Boeckh M.J., Ito J.I., Mullen C.A. (2011). Clinical practice guideline for the use of antimicrobial agents in neutropenic patients with cancer: 2010 update by the infectious diseases society of America. Clin Infect Dis.

[10] Kuderer N.M., Dale D.C., Crawford J., Cosler L.E., Lyman GH. (2006). Mortality, morbidity, and cost associated with febrile neutropenia in adult cancer patients. Cancer.

[11] Israels T., Afungchwi G.M., Klootwijk L., Njuguna F., Hesseling P., Kouya F. (2021). Fever and neutropenia outcomes and areas for intervention: a report from SUCCOUR - Supportive Care for Children with Cancer in Africa. Pediatr Blood Cancer.

[12] Assefa S., Alemayehu T., Abebe W. (2017). Factors associated with treatment outcome of pediatric cancerpatients admitted with febrile neutropenia in Tikuranbessa specialized teaching hospital, Addis Ababa, Ethiopia. Ethiop Med J.

[13] Lubwama M., Phipps W., Najjuka C.F., Kajumbula H., Ddungu H., Kambugu J.B. (2019). Bacteremia in febrile cancer patients in Uganda. BMC Res Notes.

[14] Sigurdardottir K., Digranes A., Harthug S., Nesthus I., Tangen J.M., Dybdahl B. (2005). A multi-centre prospective study of febrile neutropenia in Norway: microbiological findings and antimicrobial susceptibility. Scand J Infect Dis.

[15] Butts A.R., Bachmeier C.C., Dressler E.V., Liu M., Cowden A., Talbert J. (2017). Association of time to antibiotics and clinical outcomes in adult hematologic malignancy patients with febrile neutropenia. J Oncol Pharm Pract.

[16] Caggiano V., Weiss R.V., Rickert T.S. (2005). Linde-Zwirble WT. Incidence, cost, and mortality of neutropenia hospitalization associated with chemotherapy. Cancer.

[17] Cupp J., Culakova E., Poniewierski M.S., Dale D.C., Lyman G.H., Crawford J. (2018). Analysis of factors associated with in-hospital mortality in lung cancer chemotherapy patients with neutropenia. Clin Lung Cancer.

[18] Dulisse B., Li X., Gayle J.A., Barron R.L., Ernst F.R., Rothman K.J. (2013). A retrospective study of the clinical and economic burden during hospitalizations among cancer patients with febrile neutropenia. J Med Econ.

[19] Talcott J.A., Finberg R., Mayer R.J., Goldman L. (1988). The medical course of cancer patients with fever and neutropenia. Clinical identification of a low-risk subgroup at presentation. Arch Intern Med.

[20] Vanderpuye V., Yarney J., Beecham K. (2010). Management of febrile neutropenia in patients receiving chemotherapy for solid tumors: a retrospective study of twenty cases from the radiotherapy centre, Accra, Ghana. West Afr J Med.

[21] Poon L.M., Jin J., Chee Y.L., Ding Y., Lee Y.M., Chng W.J. (2012). Risk factors for adverse outcomes and multidrug-resistant Gram-negative bacteraemia in haematology patients with febrile neutropenia in a Singaporean university hospital. Singapore Med J.

[22] Zhou Y.P., Jin J., Ding Y., Chee Y.L., Koh L.P., Chng W.J. (2014). Direct costs associated with febrile neutropenia in inpatients with hematological diseases in Singapore. Support Care Cancer.

[23] Boccia R., Glaspy J., Crawford J., Aapro M. (2022). Chemotherapy-induced neutropenia and febrile neutropenia in the US: A beast of burden that needs to be tamed?. Oncologist.

[24] Rosa R.G., Goldani L.Z. (2014). Factors Associated with Hospital Length of Stay among Cancer Patients with Febrile Neutropenia. PLOS ONE.

[25] Klastersky J., Ameye L., Maertens J., Georgala A., Muanza F., Aoun M. (2007). Bacteraemia in febrile neutropenic cancer patients. Int J Antimicrob Agents.

[26] Viscoli C., Bruzzi P., Castagnola E., Boni L., Calandra T., Gaya H. (1994). Factors associated with bacteraemia in febrile, granulocytopenic cancer patients. The International Antimicrobial Therapy Cooperative Group (IATCG) of the European Organization for Research and Treatment of Cancer (EORTC). Eur J Cancer.

[27] Cattaneo C., Antoniazzi F., Casari S., Ravizzola G., Gelmi M., Pagani C. (2012). P. aeruginosa bloodstream infections among hematological patients: an old or new question?. Ann Hematol.

[28] Kern W.V., Andriof E., Oethinger M., Kern P., Hacker J., Marre R. (1994). Emergence of fluoroquinolone-resistant Escherichia coli at a cancer center. Antimicrob Agents Chemother.

[29] Kang C.I., Song J.H., Chung D.R., Peck K.R., Yeom J.S., Son J.S. (2012). Bloodstream infections in adult patients with cancer: clinical features and pathogenic significance of Staphylococcus aureus bacteremia. Support Care Cancer.

[30] Ghosh I., Raina V., Kumar L., Sharma A., Bakhshi S., Thulkar S. (2012). Profile of infections and outcome in high-risk febrile neutropenia: experience from a tertiary care cancer center in India. Med Oncol.

